# A potential role for the clathrin adaptor GGA in *Drosophila *spermatogenesis

**DOI:** 10.1186/1471-2121-12-22

**Published:** 2011-05-20

**Authors:** Jennifer Hirst, Jenny Carmichael

**Affiliations:** 1 University of Cambridge, Cambridge Institute for Medical Research, Cambridge CB2 0XY, UK; 2 University of Cambridge, Department of Genetics, Cambridge CB2 3EH, UK

**Keywords:** clathrin, adaptor, GGA, AP-1, spermatogenesis, Drosophila, acroblast, spermatids, spermatocytes

## Abstract

**Background:**

GGAs (Golgi-localised, γ-ear containing, ADP ribosylation factor-binding) are a family of clathrin adaptors that sort a number of biologically important transmembrane proteins into clathrin-coated vesicles. Knockout and knockdown studies to determine GGA function are confounded by the fact that there are 3 GGA genes in mammalian cells. Thus *Drosophila melanogaster *is a useful model system to study tissue expression profiles and knockdown phenotypes as there is a single GGA ortholog.

**Results:**

Here we have quantified protein expression in *Drosophila *and show that there is >3-fold higher expression of GGA in male flies relative to female flies. In female flies the majority of GGA expression is in the head. In male flies GGA is not only expressed at high levels in the head but there is a gender specific increased expression which is due to the abundant expression of GGA in the testes. Using a highly specific antibody we have localised endogenous GGA protein in testes squashes, and visualised it in somatic and germ line cells. We show that GGA is expressed during multiple stages of sperm development, and co-stains with a marker of the trans-Golgi Network. This is most striking at the acroblast of early spermatids. In spite of the high expression of GGA in testes, knocking down its expression by >95% using transgenic RNAi fly lines did not affect male fertility. Therefore spermatogenesis in the male flies appears to progress normally with <5% GGA, most likely because alternative adaptors may be able to substitute partially or completely for the function of GGA. We also identify 'cueball' as a novel cargo for GGA, and mutants of cueball have been shown to have a male sterility phenotype.

**Conclusion:**

In *Drosophila *we have uncovered a potential role for GGA in the testes of male flies. The gender specific higher expression of GGA, its specific enrichment in testes and its localisation to developing spermatocytes and at the acroblast of spermatids supports a role for GGA function in *Drosophila *spermatogenesis, even though spermatogenesis still occurs when GGA expression is depleted to <5% of control.

## Background

GGAs (Golgi-localised, γ-ear containing, ADP ribosylation factor-binding) and AP (adaptor protein)-1 are both clathrin adaptor proteins that function in the intracellular sorting of a number of biologically important transmembrane proteins between the Golgi and endocytic pathway. Where the GGAs are monomeric adaptors, AP-1 is a heterotetramer of 4 subunits (γ, β1, μ1, and σ1; [1]). Both GGAs and AP-1 are evolutionarily conserved from yeast to mammals. In mammals there are three GGA genes giving rise to GGA1, GGA2 and GGA3, and multiple isoforms of the AP-1 subunits. In contrast in *Drosophila melanogaster *there is only a single GGA ortholog, and only one isoform of each AP-1 subunit.

The function of GGAs has been elucidated largely by knockout studies in yeast [2-4], and RNAi knockdowns in mammalian [5,6] and *Drosophila *tissue culture cells [6,7]. From these studies and others it is clear that GGAs function to sort a number of proteins for incorporation into clathrin-coated vesicles, including sortilin, cation-independent mannose 6-phosphate receptor (CIMPR), cation-dependent mannose 6-phosphate receptor, sorLA, LDL receptor-related proteins (LRP), β-amyloid cleavage enzyme (BACE1), insulin-responsive aminopeptidase and stabilin-1 [[6], and references therein]. GGAs sort cargo by recognition of an acidic dileucine (DXXLL; where D is aspartic acid, X is any amino acid and L is leucine) motif usually at the C-terminus of the cytoplasmic tails. In addition to binding GGAs, many of these cargo proteins contain sorting signals for binding other adaptors. CIMPR is the best studied cargo protein in mammalian cells and it has a complex intracellular trafficking itinerary between the Golgi and endosomes. To facilitate its sorting it contains numerous sorting signals, which are recognised by AP-1 and GGA (amongst others) [8]. The only known GGA cargo in *Drosophila *is lysosome enzyme receptor protein (LERP), which is the functional equivalent of CIMPR, and like CIMPR contains both GGA and AP-1 clathrin adaptor sorting motifs [9].

The functional inter-relationship of GGAs and AP-1 has been actively debated. Both have been localised to the trans-Golgi Network (TGN) and endosomal membranes. AP-1 was shown to contain a binding site for GGAs, and this suggested the possibility that the role of GGAs may be to 'hand-on' cargo to AP-1 [10]. However, the observation that in yeast GGAs and AP-1 behave differently in the sorting of cargo in cell-free assays [2], and the visualisation in *Drosophila *Dmel2 cells of distinct populations of clathrin-coated vesicles that contain only GGA or AP-1 [6] suggests that they can function independently of each other. However, these results do not exclude either GGAs acting with AP-1 or GGAs acting independently of AP-1, nor have these studies addressed how or where GGAs function in a complex multicellular organism. For AP-1 it is clear that it plays a critical role in development since knockouts of γ adaptin [11] or μ1A [12] in mice are embryonic lethal, and knockout of μ1A in *Caenorhabditis elegans *show reduced viability [13]. For GGAs, functional studies have been hampered by redundancy between the three mammalian GGAs. From the limited information on GGA mRNA tissue expression we know that all three mammalian GGAs are ubiquitously expressed [[14], and SymAtlas http://biogps.gnf.org/]. At the protein level GGA3 expression has been shown to vary dramatically between different human tissues with expression of both isoforms (long and short) of GGA3 in the brain [15]. In addition, both GGA1 and GGA3 have been shown to play a role in the sorting of BACE1 in the brain [16,17], though the specific contribution of each GGA to BACE1 sorting is unclear. In *Drosophila*, a search of FlyAtlas (http://flybase.org/) reveals that GGA message is found ubiquitously, and at nearly all stages of larval and adult development. To date, there is no information regarding the tissue distribution of GGA protein expression in *Drosophila*.

Here we show that there is a gender specific higher expression of GGA in male *Drosophila*, and show that both GGA and AP-1 are highly expressed in *Drosophila *testes. In testes squashes both GGA and AP-1 co-localise with a marker of the TGN, most strikingly at the acroblast in developing spermatids. We also investigate GGA function in spermatogenesis using RNAi transgenic flies.

## Results

### GGA and AP-1 expression in *Drosophila*

In a previous study we raised antibodies against *Drosophila *GGA and AP-1, which allowed us to localise both endogenous adaptors in *Drosophila *tissue culture cells [6]. Here we investigated endogenous GGA and AP-1 localisation in whole flies to elucidate where these clathrin adaptors may be exerting their function.

**Figure 1:**
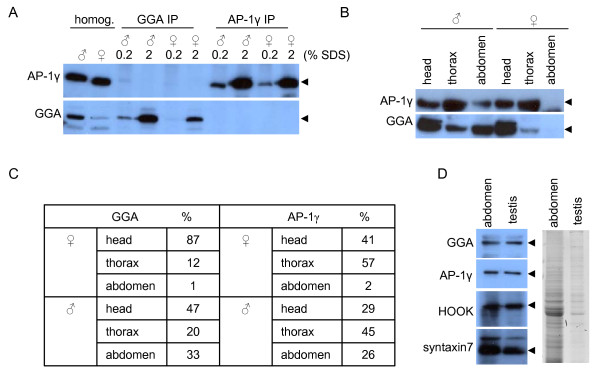
**GGA and AP-1 expression in *Drosophila***. **(A) **The relative expression of endogenous GGAs and AP-1 in male or female flies was determined in whole fly homogenates (homog.) or by immunoprecipitations (IP). The final elutions were done with either 0.2% SDS (0.2) or more harshly with 2% SDS (2). Note that AP-1 is expressed in similar amounts, but GGA is expressed >3 fold higher in the males. **(B) **Male or female flies were dissected into head, thorax and abdomen, Western blotted for AP-1 or GGA, and quantitated in **(C)**. Note that GGA is highly expressed in male abdomens. **(D) **The relative expression of GGA and AP-1 were compared in the testes of 5 flies compared to 5 abdomens. Coomassie gel shows the relative protein loading.

Western blots from whole fly lysates demonstrated that GGA was expressed at higher levels in male flies than in female flies (Figure 1A), where AP-1 was expressed at similar levels in males and females. We confirmed the relative differences in GGA expression levels using immunoprecipitations, and quantification revealed that there was >3 fold more GGA in the male flies relative to the females. In contrast there were similar levels of AP-1 in the males as the females. This led us to investigate the relative expression of GGA in dissected head, thorax and abdomens of female and male flies. Figure 1B and 1C shows that the majority of GGA expression in the females is in their heads (87% of total) with very little in the abdomens (1% of total), whereas in the males there are relatively similar levels of GGA in male heads (47% of total) compared to abdomens (33% of total). Interestingly, we also found more AP-1 in male abdomens (26% of total) relative to female abdomens (2% of total), despite there being the same amount of AP-1 overall. The abundance of GGA and AP-1 in male abdomens can be completely accounted for by their expression in dissected testes (Figure 1D).

### GGA and AP-1 Localisation in Testes Squashes

**Figure 2:**
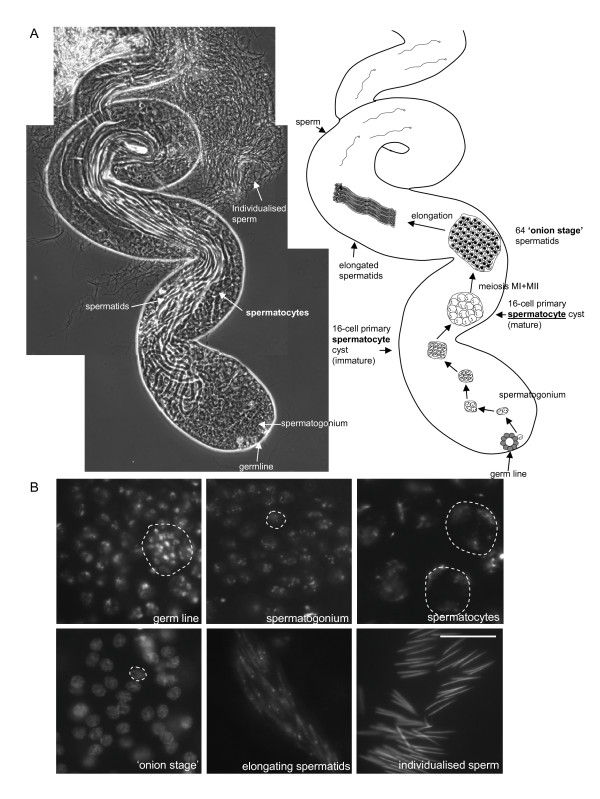
**Basic overview of *Drosophila *spermatogenesis**. **(A) **Testes were dissected from male flies and visualised by phase contrast microscopy. The overview of spermatogenesis in *Drosophila *is remarkably similar to that in mammals [29]. The process of spermatogenesis begins at the apical tip of the testes with a small number of self-renewing stem cells (germ line) that continually divide to maintain the stem cell number. These cells go through 4 synchronous mitotic amplifications with incomplete cytokinesis which result in a cyst of 16 interconnected spermatogonia. These spermatogonia (or primary spermatocytes) go through cellular growth, differentiation and is particularly characterised by high levels of gene expression. All 16 spermatocytes exit the cell growth program and undergo meiosis I and II to form a cyst of 64 inter-connected primary spermatids which have a single phase-light nucleus and a single phase-dark mitochondrial derivative (Nebenkern). In cross-section the Nebenkern has concentric rings of mitochondrial membranes that resemble an onion and give this stage its name ('onion stage'). These inter-connected haploid spermatids undergo cellular remodelling, elongating as the sperm axonemes are formed inside to form long spermatids that stretch almost the entire length of the testis. During this elongation step the spermatid flagella extend down the lumen towards the apical tip and the nuclei move down towards the basal end. The final step in spermatogenesis is a highly complex process of membrane remodelling called individualisation. In this process an actin cone assembles around the spermatid nuclei and moves synchronously from the heads to the tips of the tail, enclosing cytosol and vesicles in a cystic bulge. It is within this cystic bulge that membrane remodelling occurs to enclose each sperm axoneme to yield 64 individual sperm that are then transferred to the seminal vesicle. **(B) **Distinct stages in spermatogenesis can be easily visualised by Hoechst DNA staining. Bar, 20 μm.

**Figure 3:**
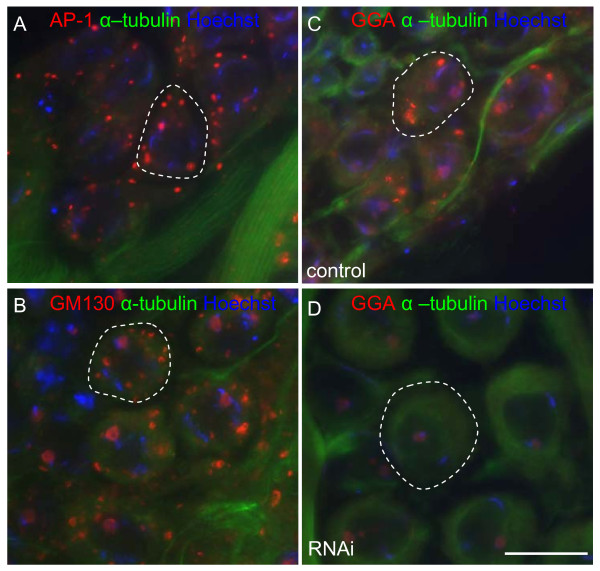
**Specific labelling of AP-1 and GGA in *Drosophila *testes**. Spermatocytes were triple labelled for α-tubulin (sperm tails and cytoplasmic microtubules), Hoechst (to determine the stage in sperm development) and either **(A) **AP-1, **(B) **GM130, and **(C) **GGA. Note that AP-1, GGA and GM130 all localise to large peripheral puncta reminiscent of Golgi mini stacks. **(D) **Spermatocytes from GGA knockdown flies were triple labelled for α-tubulin, Hoechst and GGA. Note that there is loss of the punctate cytoplasmic GGA labelling, demonstrating the specificity of the GGA antibody (the knockdown flies are further characterised in Figures 6 and 7) Bar, 20 μm

Given the abundance of GGA and AP-1 in testes as determined by Western blotting we decided to investigate their role in the testes by localising GGA and AP-1 in developing sperm. *Drosophila *testes are informative as they exhibit all stages in sperm development in a relatively simple structure. In dissected testes squashes the different stages in sperm development can be readily identified under phase contrast (Figure 2A) with characteristic patterns of Hoechst DNA staining (Figure 2B). We labelled testes squashes from control and GGA knockdown flies with antibodies against AP-1, GGA, and well characterised commercial antibodies against the cis-Golgi marker GM130, and α-tubulin (sperm tails and cytoplasmic microtubules). Using Hoechst as a guide to development stage we were able to distinguish staining of AP-1, GGA and GM130 in all stages from spermatogonia to spermatid bundles (results not shown). It was more challenging to visualise the labelling of any antibody in spermatogonia presumably because they are so small, and because antibody accessibility may be an issue. In spermatocytes we observed labelling of AP-1 (Figure 3A), GM130 (Figure 3B), and GGA (Figure 3C) in large puncta dispersed throughout the cytoplasm, and this pattern was lost in testes squashes from GGA knockdown flies (Figure 3D). In addition, we also observed localisation of AP-1 and GGA in somatic cells of the seminal vesicle (Additional file 1; note the extensive co-localisation of AP-1 and GGA with the TGN marker GCC88). We were struck by the similarity in the staining patterns for AP-1, GGA and GM130, which were reminiscent of Golgi morphology in *Drosophila *[18,19]. The main feature that distinguishes *Drosophila *Golgi from mammalian Golgi is that *Drosophila *cells contain a number of smaller Golgi stacks (that numbers 20 in S2 cells) rather than one interconnected ribbon found in mammalian cells. One exception is the acroblast in 'onion stage' spermatids, which is composed of a ribbon of numerous Golgi elements that forms the acrosome in mature sperm. Therefore the identity of the large puncta that are dispersed throughout the cytoplasm in spermatocytes and label for GM130, AP-1 and GGA are likely to be Golgi mini stacks.

**Figure 4:**
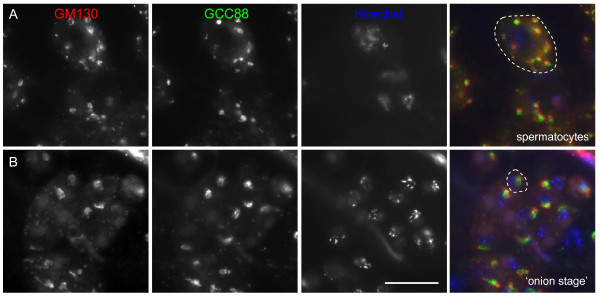
**Co staining of Golgi markers GM130 and GCC88 in *Drosophila *testes**. Testes squashes were triple labelled for GM130 (Golgi marker), GCC88 (TGN marker) and Hoechst (nuclei). Note the coincident labelling of GM130 and GCC88, seen most clearly in the large peripheral puncta in the spermatocytes **(A) **and the cone-shaped juxtanuclear acroblast in the 'onion stage' spermatids **(B)**. Note that the staining of GM130 is slightly off-set relative to GCC88 as we would expect since they are on opposed sides of the Golgi stack. Bar, 20 μm

We suspected that the localisation pattern of GGA and AP-1 was most likely to be at the TGN rather than the Golgi stack itself (see also Additional file 1). Therefore we co-stained testes squashes with antibodies against GCC88 as a marker of the trans-Golgi/TGN [20], and GM130 as a marker of the Golgi stack. GM130 and GCC88 co-localise in large puncta in spermatocytes (Figure 4A) that most likely represent Golgi mini stacks. In addition, we visualised both markers in 'onion stage' spermatids (Figure 4B). The labelling of GCC88 is slightly off-set relative to GM130 as we would expect since they are on opposite sides of the Golgi stack.

**Figure 5:**
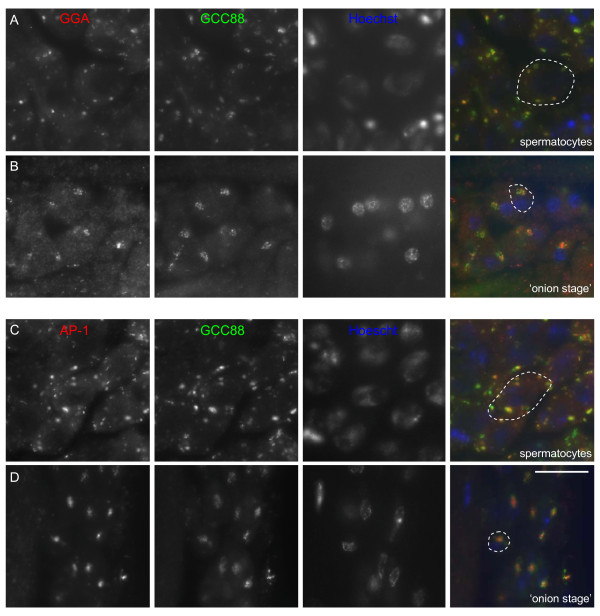
**AP-1 and GGA co-localises with the TGN marker GCC88 in *Drosophila *testes**. Testes squashes were triple labelled for either GGA or AP-1, the TGN marker GCC88 and Hoechst (nuclei). Note that GGA and AP-1 co-localises with GCC88 in spermatocytes **(A,C) **and at the acroblast in 'onion stage' spermatids **(B, D)**. Bar, 20 μm

We then co-stained testes squashes with antibodies against GCC88 and either GGA (Figure 5A,5B) or AP-1 (Figure 5C,5D). Coincident labelling of both GGA and AP-1 were observed with GCC88 in disperse Golgi mini stacks in spermatocytes (Figure 5A,5C) and coincident at the cone-shaped juxtanuclear acroblast in 'onion stage' spermatids (Figure 5B,5D). We also noted that in GGA depleted cells the GCC88 labelling appeared similar to that in control cells despite the loss of GGA expression (Additional file 2). This suggests that the acroblast, at least morphologically, is unaffected in the knockdown flies. The coincident labelling of AP-1 and GGA with GCC88 supports a role for both clathrin adaptors at the TGN in multiple stages of sperm development.

### GGA RNAi Knockdown

**Figure 6:**
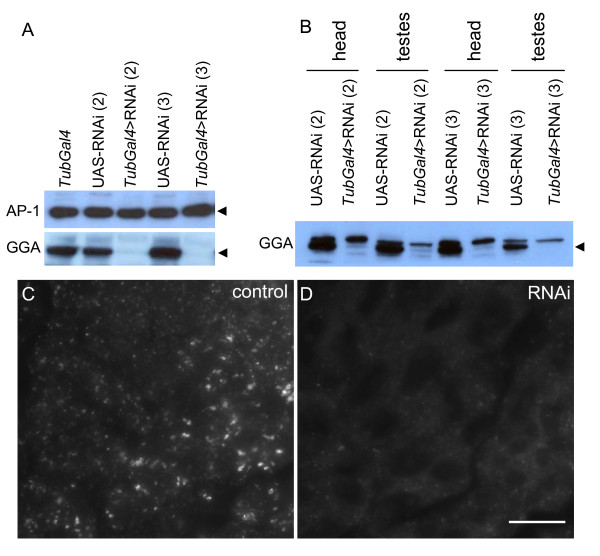
**GGA depletion in transgenic RNAi fly lines**. **(A) **GGA was depleted ubiquitously in 2 fly lines [insertions on chromosome (2) or chromosome (3)] using a *Tubulin-Gal4 *driver, and whole fly homogenates Western blotted for GGA and AP-1. In both fly lines depletion of GGA was achieved to >95%. Note that AP-1 expression does not alter in the GGA knockdown flies. **(B) **GGA knockdown flies were dissected into head and testes and the efficiency of knockdown determined by Western blotting. Note that the GGA antibody recognises a doublet, but only the bottom band is knocked down (arrowhead). Quantitation of the gels bands demonstrates that the knockdown efficiency in testes is >95%, similar to that seen in head extracts. Testes squashes from control **(C) **and GGA knockdown flies **(D) **were labelled with antibodies against GGA. Note that GGA labelling is almost completely lost in knockdown fly testes suggesting efficient GGA depletion. Bar, 20 μm.

In order to investigate whether depletion of GGA would affect spermatogenesis we used commercially available transgenic RNAi fly lines. We used two RNAi fly lines with random integration of the same RNAi construct on chromosomes 2 and 3 respectively. Knockdown was achieved with a depletion efficiency of >95% as measured in whole fly extracts, with no effect on AP-1 expression levels (Figure 6A). Similar levels of knockdown were seen in both females and males (results not shown). Since knockdown efficiencies can vary between different tissues we wanted to demonstrate that the knockdown was efficient in germ line-derived developing sperm. Therefore we isolated testes from control and knockdown flies and by Western blotting demonstrated that the efficiency of GGA knockdown was >95% (Figure 6B), which is comparable to the level seen in whole fly extracts. Furthermore we immunolabelled testes squashes from wild type and knockdown flies with antibodies against GGA and observed almost complete loss of GGA labelling in germ line derived spermatocytes and 'onion stage' spermatids (compare Figure 6C and 5D; see also Figure 3D and Additional file 2). Taken together we believe that we have demonstrated that GGA has been efficiently depleted in the developing sperm.

**Figure 7:**
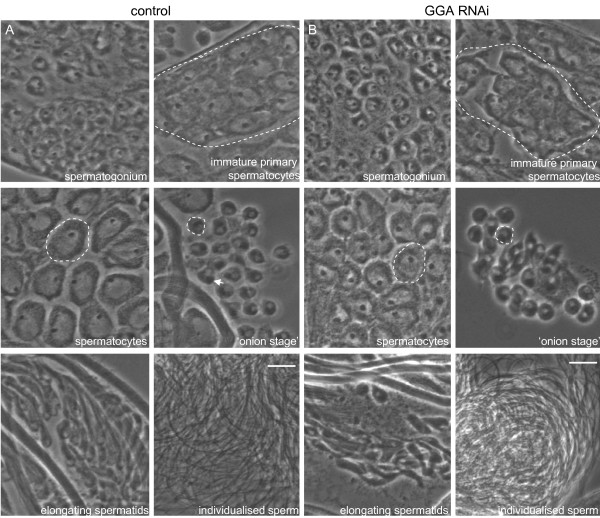
**Visualisation of all stages of spermatogenesis in GGA knockdown testes**. GGA was depleted ubiquitously using a *Tubulin-Gal4 *driver and testes squashes from male flies were compared with those from control flies. By phase contrast microscopy we observed no differences in the morphology of the stages of spermatogenesis in the GGA depleted testes. Note that all stages in spermatogenesis were visualised, culminating in individualised sperm. Bar, 20 μm.

The knockdown flies developed normally, with no gross morphological changes. We assayed the fertility of the male (and female) flies and observed no differences in the ability to produce progeny, neither in rate, number or overall development of the progeny (results not shown). We also analysed the testes from both control (Figure 7A) and GGA depleted flies (Figure 7B) and by phase contrast saw no qualitative morphological differences in the development of sperm, with both culminating in the production of individualised sperm. These sperm were clearly motile since the sperm were easily visualised when seminal vesicles were accidentally pierced during the dissection (results not shown). Given the efficiency of knockdown these results suggest that GGA depletion does not result in any measurable loss of male fertility.

### A search for novel GGA cargoes

**Figure 8:**
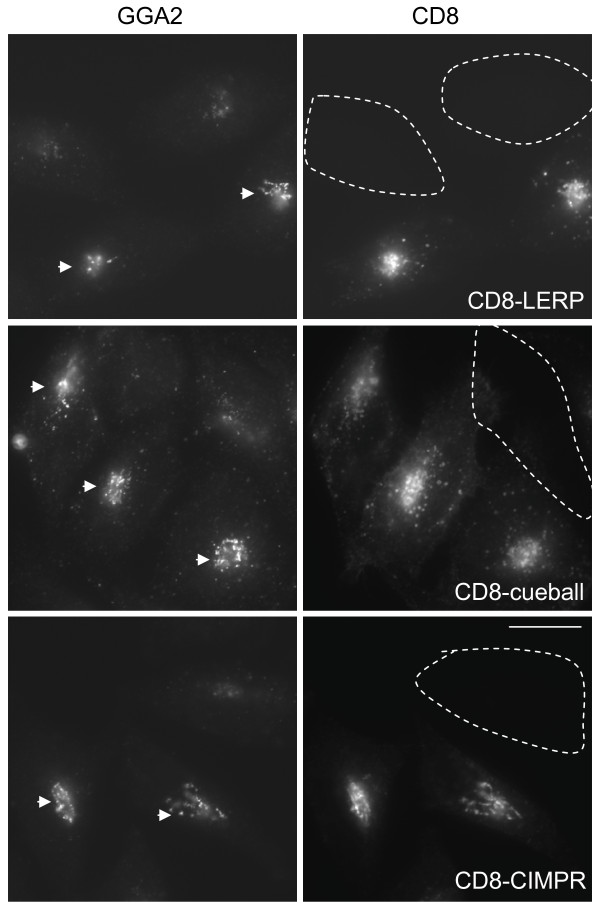
**Expression of a CD8-cueball reporter construct increases GGA2 recruitment in HeLa cells**. The cytoplasmic tails of CIMPR, LERP and cueball were expressed in a CD8 reporter system. Transient expression of CD8-CIMPR, CD8-LERP and CD8-cueball in HeLa cells resulted in the increase of GGA2 recruitment to membrane as shown by the increase in fluorescence intensity in the juxtanuclear region of the cell (arrowhead). The encircled cells highlight cells where there is no expression of CD8-constructs. Bar, 20 μm

Given the enrichment of GGA in testes we wanted to investigate what cargo proteins might require GGA for their sorting. We therefore constructed a database of cytoplasmic tails of type I transmembrane proteins and searched for those that contain a GGA binding consensus (Table 1). Of the 1700 protein entries in the database only five entries were identified with possible GGA binding motifs. Reassuringly two of these hits were different transcripts of LERP, the only known GGA cargo in *Drosophila *[9]. Of the remaining three hits the most interesting was 'cueball' because it has been shown to function in primary spermatocyte growth, where mutants in cueball lead to a male sterility phenotype [21]. By BLAST analysis of human databases cueball shows most homology to the family of proteins of low density lipoprotein receptor-related proteins (LRPs). Many members of the LRP family contain multiple sorting motifs for binding clathrin adaptors. Indeed, LRP9 has an acidic dileucine motif that acts as a dual GGA and AP-1/AP-2 binding motif [22]. Likewise, cueball also contains a potential dual acidic dileucine motif (as well as other possible sorting motifs) and therefore its trafficking may be regulated by a number of coat proteins that includes AP-1 and GGA. We have previously shown that overexpression of cargo cytoplasmic tails as a CD8-reporter construct (such as CD8-CIMPR) in HeLa cells specifically increases the recruitment of GGAs to membrane [23, and Figure 8], and can be used as a criteria to define novel bonafide GGA cargoes. Here we found that overexpression of the cytoplasmic tail of cueball (CD8-cueball) was sufficient to increase the recruitment of mammalian GGA2 (Figure 8), and supports the idea that cueball is a novel GGA cargo protein.

**Table 1:** Type I membrane proteins with GGA sorting motifs

Symbol	FB Accession	Ensembl Accession	UNIPROTKB	CG	Cytoplasmic tail
CG12576-PA	FBpp0077057	FBgn0031190	Q8IQ25 Q9VR57	CG12576-PA	...TALDSSDES**D**FE**IL**ESDDFKstop

cue-PA	FBpp0072590	FBgn0011204	Q95RU0	CG12086-PA	...KSSCKE**D**KK**IL**IHNMEDDLYstop

Lerp-PB	FBpp0084467	FBgn0051072	Q8IMR0 Q8IMR1	CG31072-PB	...ANLLLEPNGEFTESD**D**DM**LL**stop

Lerp-PA	FBpp0099603	FBgn0051072	Q8IMR0 Q8IMR1	CG31072-PA	...ANLLLEPNGEFTESD**D**DM**LL**stop

CG31150-PA	FBpp0082643	FBgn0051150	Q9VF24	CG31150-PA	...EANTPTPVPLQNSS**D**VV**VV**Astop

A database of *Drosophila *type I transmembrane proteins were searched for GGA binding consensus motifs. Five proteins were identified; only the last ~20 amino acids of the cytoplasmic tails are shown here with the amino acids that correspond to a predicted GGA sorting motif highlighted in bold.

In summary, we have shown that we can efficiently deplete GGA expression to >95% in germ line derived developing sperm, and the knockdown male flies have morphologically normal testes and they exhibit no loss of fertility. Therefore with regard to fertility male flies appear to be able to function normally in the presence of <5% GGA. In addition, we have also shown that the male flies express >3 fold higher expression of GGA relative to the females, and that GGA is abundantly expressed not only in the head of flies but also specifically in the testes in multiple stages during the process of spermatogenesis. We have also identified cueball as a novel binding partner of GGA, and this protein has important functions in the growth of primary spermatocytes. These results suggest that GGA plays a role at the TGN in multiple stages of sperm development.

## Discussion

The GGAs were first described in 2000 and since then our understanding of their role has risen exponentially. In mammalian cells the mRNA expression profiles of GGA1-3 predict that they are ubiquitously expressed [[14], SymAtlas], and several studies have identified roles for GGA1 and GGA3 in the sorting of BACE1 in the brain [16,17]. However, their function in multicellular organisms remains elusive due to the absence of animal knockout or knockdown models, and studies in mammalian cells are confounded by their functional redundancy. *Drosophila *not only provides us with a well studied model system but functional redundancy is no longer a confounding factor as there is only a single GGA ortholog. A search on FlyAtlas reveals that GGA mRNA message is found ubiquitously, and that there is moderate message at all stages of development in nearly all larval and adult fly stages. Here we have quantified GGA protein levels and show that in female flies the majority of GGA expression is in the heads (87%), suggesting that in the females the principal function of GGA is in the head. More significantly we show that the male flies express >3 fold the total level of GGA than the females, and the increase in expression is largely confined to the testes. These results suggest the possibility of two separate roles for GGA, one in the head, where the function is shared between female and male flies, and one function in the testes that is specific to the male flies. For this paper we chose to focus our attention on the role of GGA in male testes. Interestingly, the publically available information on SymAtlas suggests that there is abundant GGA1 message in testes. Given the similarities between spermatogenesis in mammals and *Drosophila *it would be interesting to determine if there is gender specific higher GGA1 protein expression in mammals.

In order to determine where GGA may be functioning in testes we performed localisation studies in germ line and somatic cells. All antibody labelling was difficult to resolve in early spermatogonia, but GGA labelling was readily observed in Golgi mini stacks in spermatocytes. Spermatocytes are significantly larger than spermatogonia, which probably make it easier to resolve the labelling, but this is also a stage when expression of many proteins involved in spermatogenesis is switched on or upregulated. We also observed the localisation of GGA at the cone-shaped acroblast of 'onion stage' spermatids. For AP-1, despite the similar levels of overall protein expression levels in males and females, like GGA, there is proportionally more AP-1 in the male abdomens compared to the females. This expression is confined to the testes, and localises to the TGN in spermatocytes and 'onion stage' spermatids. This suggests that AP-1, like GGA, may also play a role at the TGN in different stages of sperm development. Efficient vesicle traffic is necessary in order to support the massive increase in cell membrane surface area which is required for the extensive membrane remodelling to enlarge spermatocytes (25 times increase in volume), to elongate differentiating spermatids (100-fold increase in length), and the final step of individualising spermatids. It is therefore likely that membrane trafficking may play multiple important roles in these massive remodelling events. Indeed, clathrin (and more recently auxilin [24] which is a regulator of clathrin function) has been shown to play a role in the remodelling that occurs in the individualisation of spermatids [25]. The mechanism of clathrin function is not well understood, but it is possible that one of the roles of GGA (and AP-1) may be in cooperation with clathrin in intracellular sorting events during membrane remodelling.

We attempted to further elucidate the role of GGA in *Drosophila *spermatogenesis by knocking down GGA expression using transgenic RNAi fly lines. Here we used a *Tubulin-Gal4 *driver to ubiquitously knockdown GGA expression. Although knockdowns efficiencies are likely to vary between different tissues we were able to show by Western blotting of dissected testes that we achieved >95% loss of GGA protein, which was comparable to the level of knockdown we saw in fly extracts. In addition by immunolabelling of dissected testes we saw almost complete loss of GGA labelling in multiple stages of germline-derived developing sperm. Combined, these data suggest that knockdown of GGA in germline was efficient, however, in spite of this efficiency we did not observe any effects on male fertility. It is possible that the remaining <5% GGA may be sufficient for some function. However, we have also shown here that AP-1, like GGA, is also highly expressed in testes and localises to the TGN in spermatocytes and acroblast of spermatids. In addition, the family of Mint proteins have also been shown to localise at the TGN and like GGAs function in the sorting of cargo out of the TGN [26]. Therefore it seems most likely that the lack of a phenotype in our GGA depleted flies may be due to the ability of AP-1 and/or Mint to fully or partially substitute for the loss of GGA. It would therefore be interesting to analyse the effects of loss of GGA, AP-1 and/or Mint in spermatogenesis. Certainly ubiquitous knockdown of AP-1 in *Drosophila *may well be lethal (since knockout of AP-1 in mice is lethal [11,12]), however, using the GAL4/UAS system it should be possible to produce conditional tissue-specific inactivation of GGA and AP-1 in germ line. However, at present there are no male specific drivers currently available (http://flystocks.bio.indiana.edu/).

The expression and localisation of GGA and AP-1 in germ line cells supports a potential role for these adaptors in the sorting of cargo out of the TGN during spermatogenesis. What cargo might GGA be responsible for sorting in testes? As yet the only known cargo for GGA in *Drosophila *is LERP [9], which is the functional equivalent of CIMPR. LERP contains sorting signals for both adaptors and GGA, and it has been shown that AP-1 and GGA cooperate to traffic LERP between the TGN and endosomes [6,7]. However, this function is unlikely to be restricted to testes (FlyAtlas). In order to search for other potential GGA cargoes we constructed a database of type I cytoplasmic tails, searched for proteins that contain a GGA consensus binding motifs, and identified the protein 'cueball'. This was particularly intriguing given that mutants in cueball have been shown to lead to a male sterility phenotype [21]. By BLAST analysis cueball shows most homology to the mammalian family of proteins of low density lipoprotein receptor-related proteins (LRPs). Many members of the LRP family contain multiple sorting motifs for binding clathrin adaptors. Indeed, LRP9 has an acidic dileucine motif that acts as a dual GGA and AP-1/AP-2 binding motif [22]. Likewise, cueball also contains a potential dual acidic dileucine motif, and using a CD8-reporter system we have shown that cueball can increase the recruitment of mammalian GGA2, which we have shown previously is a criteria for being a bonafide GGA cargo protein [23]. Therefore cueball is likely to be a novel cargo protein for GGA, and given the presence of a dual acidic dileucine motif its trafficking may be regulated by AP-1/AP-2 as well as by GGA (amongst others such as Mints). It will be interesting to further elucidate the contribution of GGA in cueball trafficking, particularly in relation to its role in spermatocyte growth and development.

## Conclusions

In *Drosophila *we have uncovered a role for GGA in the testes of male flies. The gender specific higher expression of GGA, its enrichment specifically in testes, and its localisation in spermatocytes and particularly at the acroblast of developing spermatids supports a role for GGA in *Drosophila *spermatogenesis. We describe for the first time the knockdown of GGA in a multicellular organism and show that in terms of male fertility the flies appear to be able to function with only <5% of GGA (presumably because AP-1 and/or Mints can functionally substitute). Since spermatogenesis is remarkably similar in mammals as it is in *Drosophila*, it will be of interest to determine if GGAs (particularly GGA1) play a role in mammalian spermatogenesis.

## Methods

### Antibodies

Antibodies against *Drosophila *GGA and AP-1 were raised in-house and have been described elsewhere [6]. Other antibodies used in this study included anti-α tubulin (Sigma), anti-dGM130 (Abcam), anti-CD8 (Ancell), anti-GGA2 [4], anti-dGCC88 (S. Munro), anti-HOOK (H. Krämer), and anti-syntaxin 7 (H. Krämer).

### Fly Husbandry

GGA (FlyBase entry ***Dmel\Gga ***CG3002) RNAi flies were obtained from the Vienna *Drosophila *RNAi Center (http://stockcenter.vdrc.at). The library comprises 22,247 transgenic *Drosophila *strains with 88% coverage of the *Drosophila *genome [27] constructed in the host strain *w*^*1118*^. The GGA RNAi transgene was constructed by cloning a short gene fragment as an inverted repeat, and expressed using the *UAS-GAL4 *system in order to enable conditional inactivation of gene function in specific tissues. Two GGA transgene fly lines were established in which the insertion sites were mapped to chromosome 2 ('viable') and chromosome 3 ('lethal', and therefore maintained over a *TM6B *balancer). All controls were done in an isogenic background, and ubiquitous gene inactivation was induced using a *Tubulin-Gal4 *driver obtained from Bloomington Stock Center (http://flystocks.bio.indiana.edu/). Fly stocks were raised and maintained at 25°C and grown on standard cornmeal-yeast-glucose medium.

### Testes Squashes and Immunofluorescence

Intact testes from male flies were dissected in phosphate buffered saline (PBS) and each of the two testes placed in a 3 μl drop of PBS at opposite corners of an 18 × 18 mm cover slip. The testes were carefully cut using a sharp tungsten needle to release contents, sandwiched between a microscope slide and then flash frozen in liquid nitrogen. The cover slips were removed and then the slides rapidly fixed in ice cold methanol for 5 mins at -20°C, and then acetone for 1 mins at -20°C. The samples were rehydrated in PBS 1% Triton X-100 for 10 mins, and blocked in PBS-BSA (1% BSA) for 45 mins and then labelled with primary antibody containing Hoechst (final concentration 0.5 μg/ml) under a 22 × 22 mm cover slip at 4°C in a humid chamber overnight. The slides were washed with PBS, and then incubated with appropriate secondary antibodies diluted in PBS-BSA for 1 h at room temperature. Finally slides were washed in PBS and mounted in mounting media and imaged with a Zeiss Axiovert 200 inverted microscope using a Zeiss Plan Achromat 63x oil immersion objective, a Hamamatsu ORCA-ER2 camera, and Improvision OPENLAB software.

### Western Blotting and Immunoprecipitations

For Western blotting, flies were separated into males and females (dissected into head, thorax, abdomen and testes where appropriate) frozen on dry ice and then mechanically homogenised in 10 μl/fly RIPA buffer (50 mM Tris, 150 mM NaCl, 0.1% SDS, 0.5% Na.Deoxycholate, 1% Triton X-100, 2mM EDTA and protease inhibitor cocktail) using a microfuge pestle. The sample was sonicated, spun at 13,000 g for 5 mins and then 4x sample buffer added.

For immunoprecipitations 80 male or female flies were lysed in PBS/0.5% NP40, precleared with Protein A-Sepharose, and then incubated with primary antibody for 1 h at 4°C, followed by Protein A-Sepharose for 45 mins at 4°C. The beads were washed repeatedly with PBS/0.5% NP40, a final wash in PBS and then eluted in mild conditions 50 mM Tris pH7.4/0.2% SDS or more harshly in 50 mM Tris pH7.4/2% SDS. The elutants were acetone precipitated and then resuspended in sample buffer. Western blots were quantified using IMAGE J software.

### Cargo Identification

GGAs are known to sort a number of type I membrane proteins by recognition of acidic dileucine motifs at the C-terminus of the cytoplasmic tails. We searched a publicly available database of cytoplasmic tail sequences to identify GGA cargo [28]. Using an expanded search criteria to look for proteins with slight variations of the consensus (D\w\w[LIMV][LIMV]\w{0,3}$, and D\w\w[LI][LI]\w{0,20}$) we were able to identify most of the known type I GGA cargoes including SorLA, BACE1, stabilin-1, LRP12, LRP10, CIMPR, CDMPR (results not shown). Having validated the search criteria in the human database we performed a similar search in a database that we constructed of 1700 *Drosophila *type I cytoplasmic tails, and identified 5 type I membrane proteins that contain a GGA binding consensus. Two of the hits are different transcripts of LERP which is the only known cargo for GGA in *Drosophila *and serves to validate the search criteria and the database construction, one has unknown function (CG12576), one protein is predicted to have lipid transporter activity (CG31150), and the most interesting hit is 'cueball' (CG12086) which shares homology to the family of LRP proteins and contains a dual GGA and adaptor sorting motif. According to FlyAtlas all 5 are expressed ubiquitously, with expression in testes. We have previously shown that cargo can regulate the recruitment of GGA to membrane; expression of the cytoplasmic tails of known GGA cargo proteins (CIMPR and sortilin) in a CD8-reporter construct specifically increases the recruitment of GGAs in HeLa cells [23], and expression of the cytoplasmic tail of the only known *Drosophila *GGA cargo in a GFP-reporter construct (GFP-LERP) similarly increases GGA recruitment in Dmel2 cells [6]. We therefore cloned the cytoplasmic tail of LERP and cueball into the CD8-reporter system in pIRESneo2 using AflII and Not1 cloning sites located just upstream of the transmembrane domain of CD8, and expressed these constructs transiently in HeLa cells and monitored the recruitment of GGA to membranes.

## Authors' contributions

JC performed all the fly manipulations, participated in the design of experiments and interpretation of data. JH performed all other experiments, participated in the design of experiments, the interpretation of data, and the writing of the manuscript. Both authors read and approved the final manuscript.

## Supplementary Material

Additional file 1 **AP-1 and GGA localisation in somatic cells of the seminal vesicle**. Testes squashes were triple labelled for either AP-1 or GGA, and the TGN marker GCC88 and Hoechst. Note the coincidence of labelling between AP-1/GGA and GCC88 in large puncta dispersed throughout the cytoplasm. These structures most likely correspond to Golgi mini stacks. Bar, 20 μm Click here for file

Additional file 2 **Acroblast formation in GGA RNAi depleted cells**. GGA was depleted ubiquitously using a *Tubulin-Gal4 *driver, and testes squashes from knockdown male flies **(A) **and control male flies **(B) **were triple labelled for GGA, the TGN marker GCC88 and Hoechst. Note that the acroblast in the 'onion stage' spermatid stage appears morphologically unaffected where GGA expression is depleted. Bar, 20 μm Click here for file
